# Efficacy and Outcomes of Para-Annular Plication in Mitral Valve Repair via Right Mini-Thoracotomy

**DOI:** 10.7759/cureus.67623

**Published:** 2024-08-23

**Authors:** Kenichi Morimoto, Shigeto Miyasaka, Rikuto Nii, Yosuke Ikeda

**Affiliations:** 1 Cardiovascular Surgery, Tottori Prefectural Central Hospital, Tottori, JPN

**Keywords:** cardiac surgical procedures, mitral valve surgery, mitral valve repair, minimally invasive cardiac surgery, left atrial plication

## Abstract

Purpose: We aim to assess the efficacy and safety of left atrial plication (LAP), particularly para-annular plication, using a right mini-thoracotomy approach.

Methods: Among 90 mitral valve repair (MVr) procedures performed at our institution between 2016 and 2023, 16 left atrial plication cases for left atrial enlargement (diameter: >50 mm) were assessed; nine cases underwent median sternotomy (conventional) (Group C), and seven cases underwent minimally invasive cardiac surgery (MICS) (Group M). The surgical protocol involved mitral valve repair via a right-sided left atrial approach, incorporating para-annular plication to suture the posterior wall. The mean follow-up duration was 3.3±2.4 years.

Results: Mortality within 30 days of surgery or during hospitalization did not occur. Postoperative complications included one case in each group that required reoperation for hemorrhage originating extraneously in the left atrium. Postoperative echocardiographic assessments revealed a comparable reduction in left atrial diameter (C/M: 80.3±7.0/80.7±14.6%; p=0.94), left atrial volume index (55.6±19.3/68.3±34.1%; p=0.36), and aorto-mitral angle (AMA) enlargement (113.8±7.3/107.5±12.2%; p=0.22). The three-year survival rate (88.9%/75.0%; p=0.33) was comparable between groups.

Conclusion: The synergistic utilization of left atrial plication with para-annular plication via right mini-thoracotomy can enhance the postoperative outcomes of mitral valve repair.

## Introduction

Left atrial (LA) enlargement frequently occurs in patients with valvular heart disease. In mitral valvular pathologies, the LA commonly undergoes dilation due to augmented LA pressure and volume, which has been postulated to induce irreversible ramifications on atrial fibrillation and respiratory physiology. The efficacy of an adjunctive intervention, known as "left atrial plication (LAP)," has been reported in the literature [1-3].

Conversely, in the contemporary landscape of minimally invasive cardiac surgery (MICS), mitral valve repair (MVr) via right mini-thoracotomy for mitral regurgitation (MR) has also demonstrated efficacy [4,5]. However, there is no evidence regarding the usefulness of concomitant LAP during mitral valve surgery via the right mini-thoracotomy approach.

Therefore, this study aimed to assess the efficacy and safety profile of LAP, particularly focusing on para-annular plication in the context of MICS-MVr. The study pioneers the use of the mini-thoracotomy approach for LA plication, presenting a novel technique to the field and demonstrating its unique advantages.

## Materials and methods

Patient cohort

Among the cohort of individuals who underwent MVr for MR at the Department of Cardiovascular Surgery, Tottori Prefectural Central Hospital, from 2016 to 2023, the study inclusion criteria encompassed 16 patients with an enlarged LA (defined as an LA diameter > 50 mm) who underwent concurrent LAP. Patients with an ischemic MR pathology were excluded from the study. A retrospective analysis of nine patients who underwent median sternotomy (classified as the conventional approach) (Group C) and seven patients who underwent MICS (Group M) was performed.

Prior to the retrospective analysis and reporting of findings, explicit patient consent was obtained. This study was conducted in accordance with the guidelines of the Institutional Review Board of the Tottori Prefectural Central Hospital, ensuring adherence to ethical standards (approval number: 2024-05).

Clinical data

A comprehensive array of clinical parameters was meticulously extracted from the medical repository, which included demographic attributes, such as age and sex; anthropometric indices, including body mass index; preoperative comorbidities, including hypertension, chronic obstructive pulmonary disease (COPD), diabetes mellitus, hypercholesterolemia, chronic kidney disease, and hemodialysis; New York Heart Association classification; Japan SCORE II; laboratory investigations; echocardiographic evaluations; details of the surgical intervention; operative duration; duration of cardiopulmonary bypass (CPB); and duration of cardiac arrest.

Echocardiography

**Figure 1:**
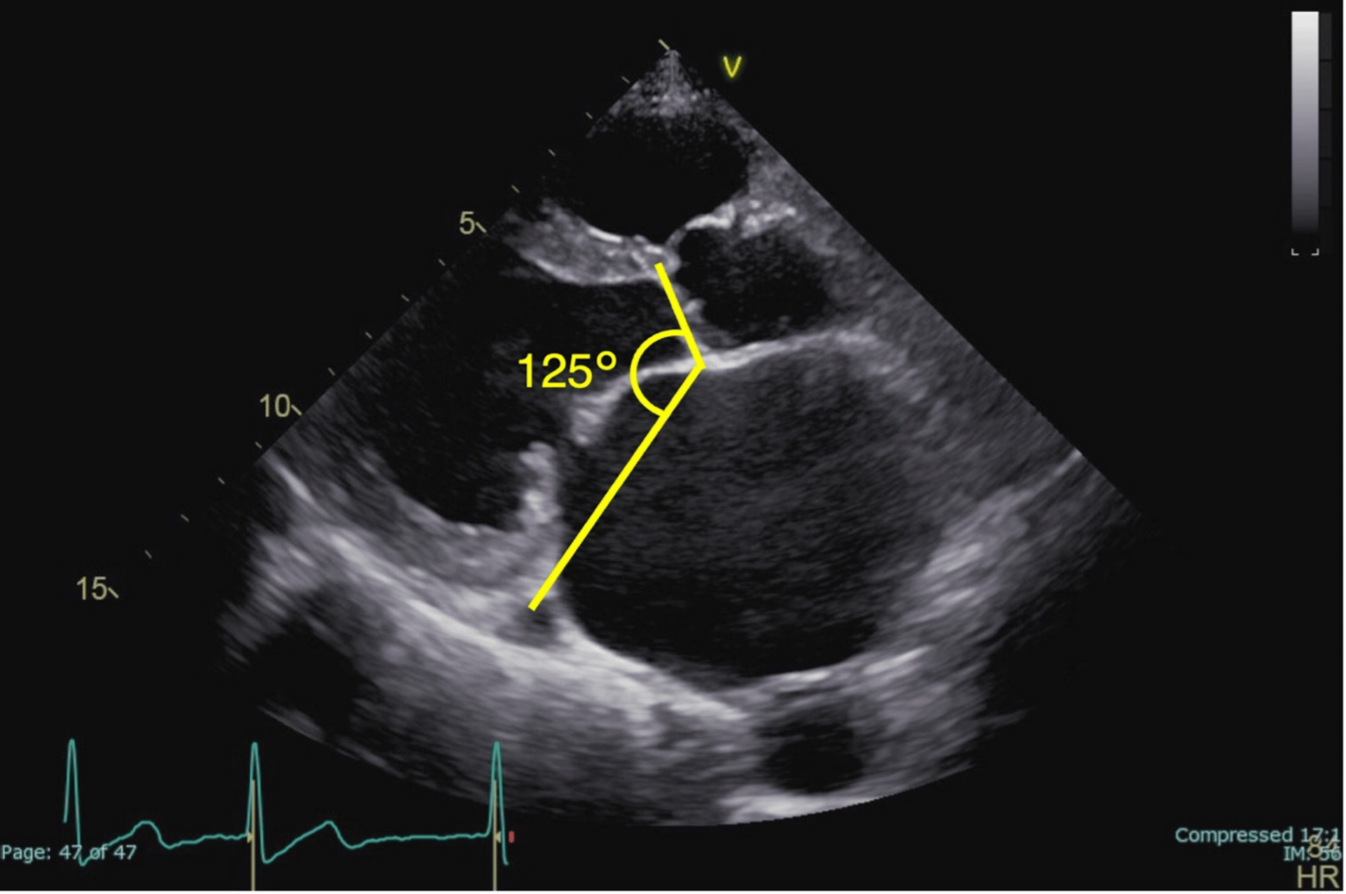
Measurement of the AMA on transthoracic echocardiography AMA: aorto-mitral angle

Preoperative transthoracic echocardiography (TTE) was performed in all patients. Left ventricular end-diastolic dimension (LVDd), left ventricular end-systolic dimension (LVDs), and left atrial (LA) dimension were assessed using the paraventricular left ventricular (LV) long-axis image, whereas the LA volume was quantified using the apical quadrant image. In addition, the LA volume index (LAVi) was calculated. The aorto-mitral angle (AMA), defined as the angle of intersection between the mitral annulus ring and the aortic valve plane at end-diastole, was gauged from digitally recorded images on parasternal long-axis views, as depicted in Figure 1. Postoperative TTE was conducted within 7-10 days post-surgery, followed by subsequent assessments at intervals of six months and one year.

Surgical procedure

In Group C, patients were placed in the supine position and underwent a median sternotomy. Conversely, in Group M, a right mini-thoracotomy was performed through a 6-cm incision situated lateral to the midclavicular line. CPB in Group C involved cannulation of the ascending aorta and the right atrium. Conversely, in Group M, femoral artery/vein cannulation was performed following a groin incision. When deemed necessary, owing to inadequate cardiac collapse or concurrent tricuspid valve surgery, decannulation of the superior vena cava (SVC) was performed. After ensuring satisfactory systemic perfusion with CPB, the ascending aorta was cross-clamped via the transverse sinus, facilitating the delivery of antegrade crystalloid cardioplegia through a cardioplegia catheter. Subsequently, access to the LA was established through an atrial incision with retraction assistance. A right-sided left atriotomy approach was consistently employed in all cases. In Group M, the procedure was performed under direct vision and thoracoscopic assistance.

**Figure 2:**
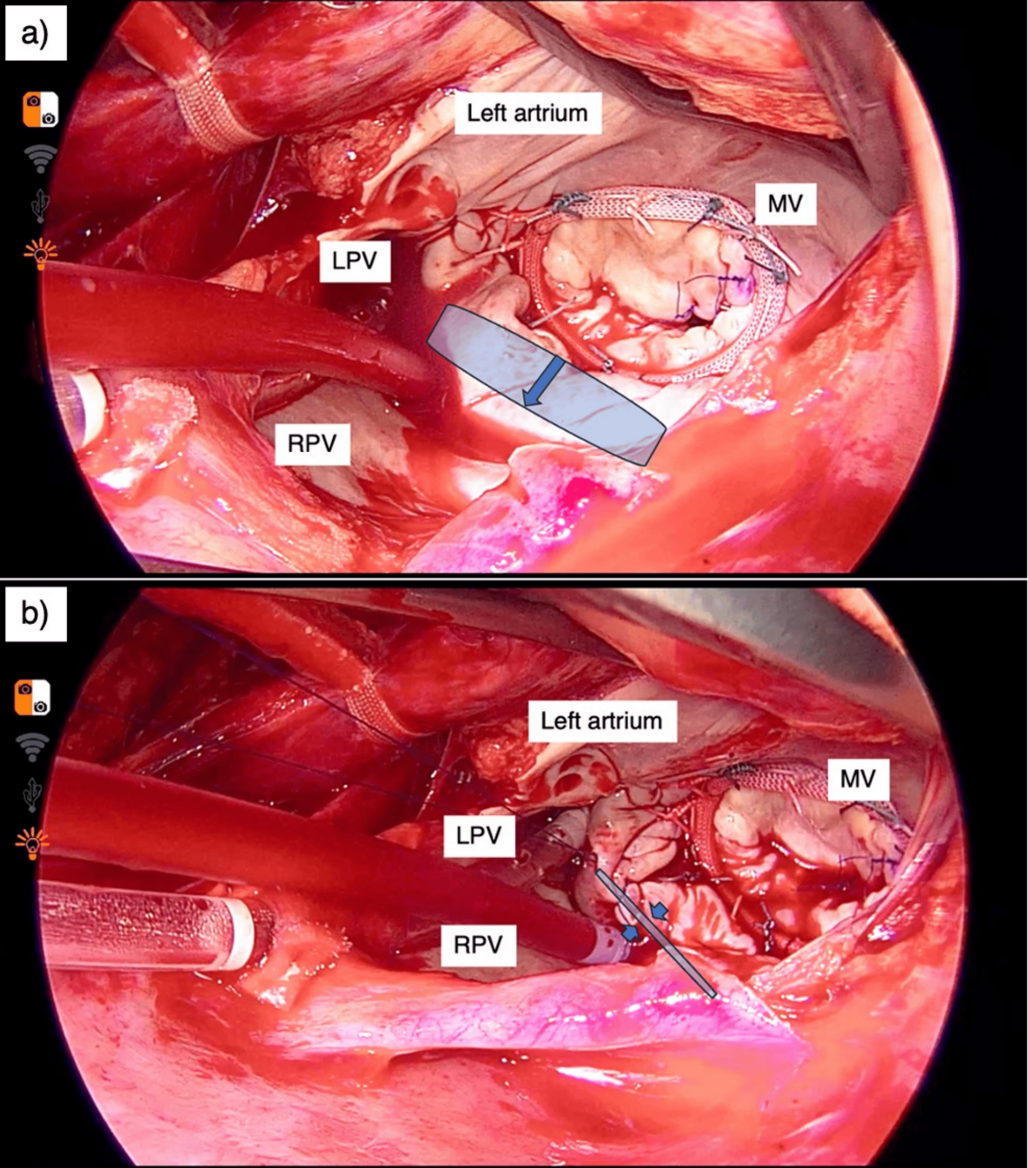
Left atrial plication through right mini-thoracotomy A right mini-thoracotomy was performed through an approximately 6-cm skin incision lateral to the midclavicular line. After confirming secure systemic perfusion with cardiopulmonary bypass, the ascending aorta was cross-clamped with a Cygnet clamp (Vitalitec, Plymouth, MA) placed through the transverse sinus. Antegrade crystalloid cardioplegia was induced using a cardioplegic catheter. The left atrium was entered through an atrial incision, and a retractor was positioned. The procedure was performed under direct thoracoscopic assistance. a: The area marked in blue represents the left atrial posterior wall enlarged in the direction of the arrow. b: The left atrial posterior wall was plicated with a width of 4-5 cm using a double layer of 4-0 polypropylene sutures. MV: mitral valve, LPV: left pulmonary vein, RPV: right pulmonary vein

Para-annular plication, entailing suturing of the posterior LA wall between the mitral valve and the right and left inferior pulmonary veins with two layers of continuous sutures using 4-0 monofilament, was performed adjunctively during MVr using an annuloplasty ring. Closure of the LA appendage was separately performed within the luminal cavity using continuous sutures with a 4-0 monofilament thread, as illustrated in Figure 2.

Statistical analyses

Categorical variables were presented as absolute values and percentages and were compared using the chi-square test. Continuous variables were expressed as mean±standard deviation and analyzed using Student's t-tests. Survival analyses were conducted using the Kaplan-Meier method, and intergroup disparities were assessed using the log-rank test. Statistical significance was defined as a p-value of <0.05. All statistical computations were conducted using EZR version 1.66 (Saitama Medical Center, Jichi Medical University, Saitama, Japan), a user-friendly graphical interface for R (R Foundation for Statistical Computing, Vienna, Austria) tailored to accommodate the statistical functions commonly used in biostatistics.

## Results

Patient characteristics

Table 1 shows the patients' preoperative characteristics. The mean age (Group C: 76.0±5.8 years, Group M: 74.9±8.1 years; p=0.73), LV ejection fraction (EF) (C: 68.4±6.6%, M: 62.8±17.8%; p=0.34), brain natriuretic peptide (BNP) levels (C: 420±199 pg/mL, M: 304±430 pg/mL; p=0.67), and Japan SCORE II scores (C: 5.6±2.8%, M: 4.4±5.3%; p=0.51) were not significantly different between the two groups (Table 1). Furthermore, there were no instances of active infective endocarditis, emergent cases, or reoperations in the entire cohort of 16 patients.

**Table 1:** Baseline patient characteristics Data are presented as numbers (%) unless otherwise indicated. p-values less than 0.05 were considered statistically significant. COPD: chronic obstructive pulmonary disease, NYHA: New York Heart Association, MICS: minimally invasive cardiac surgery, Group C: conventional group, Group M: MICS group

	All (n=16)	Conventional group (n=9)	MICS group (n=7)	p-value (Group C versus Group M)
Age, years	75.5±6.3	76.0±5.8	74.9±8.1	0.73
Male, number (%)	9 (56.3)	5 (55.6)	4 (57.1)	1
Body surface area, m^2^	1.45±0.21	1.43±0.16	1.46±0.26	0.761
Hypertension, number (%)	14 (87.5)	7 (77.8)	7 (100)	0.475
COPD, number (%)	4 (25)	2 (22.2)	2 (28.6)	1
Diabetes mellitus, number (%)	2 (12.5)	1 (11.1)	1 (14.3)	1
Hypercholesterolemia, number (%)	5 (31.3)	2 (22.2)	3 (42.9)	0.596
Chronic kidney disease, number (%)	10 (62.5)	4 (44.4)	6 (85.7)	0.145
Hemodialysis, number (%)	2 (12.5)	1 (11.1)	1 (14.3)	1
NYHA				
I, number (%)	1 (6.25)	0 (0)	1 (14.3)	0.438
II, number (%)	8 (50.0)	4 (44.4)	4 (57.1)	1
III, number (%)	7 (43.8)	5 (55.6)	2 (28.6)	0.358
IV, number (%)	0 (0)	0 (0)	0 (0)	
Japan SCORE II, %	5.1±3.7	5.6±2.8	4.4±5.3	0.507
Serum albumin, g/dL	3.9±0.4	4.0±0.4	3.9±0.4	0.533
Brain natriuretic hormone, pg/mL	369±288	420±199	304±430	0.665

Surgical procedure

Table 2 summarizes the surgical interventions. All patients underwent MVr using an annuloplasty ring. Mitral valve annuloplasty alone, necessitating no leaflet intervention, was performed in two (22%) and four (57%) patients in Group C and Group M, respectively (p=0.30). Concomitant procedures included pulmonary vein isolation in five (56%) patients and one (14%) patient (p=0.15), respectively, and tricuspid valve annuloplasty in six (67%) and three (43%) patients (p=0.62), respectively. All patients had atrial fibrillation and underwent LA appendage closure. No significant difference in the total operative time (C: 331±30 minutes, M: 297±99 minutes; p=0.27) and duration of cardiac arrest (C: 120±45 minutes, M: 126±34 minutes; p=0.77) was observed between the two groups.

**Table 2:** Surgical procedure Data are presented as numbers (%) unless otherwise indicated. p-values less than 0.05 were considered statistically significant. MICS: minimally invasive cardiac surgery, Group C: conventional group, Group M: MICS group

	All (n=16)	Conventional group (n=9)	MICS group (n=7)	p-value (Group C versus Group M)
Left atrial approach, number (%)	16 (100)	9 (100)	7 (100)	1
Only mitral valve annuloplasty, number (%)	6 (37.5)	2 (22.2)	4 (57.1)	0.302
New chordal replacement, number (%)	8 (50)	5 (55.6)	3 (42.9)	1
Left atrial appendage closure, number (%)	16 (100)	9 (100)	7 (100)	1
Tricuspid valve annuloplasty, number (%)	9 (56.3)	6 (66.7)	3 (42.9)	0.615
Pulmonary vein isolation, number (%)	6 (37.5)	5 (55.6)	1 (14.3)	0.145
Full ring, number (%)	16 (100)	9 (100)	7 (100)	1
Ring size, mm	30.6±2.3	30.9±1.9	30.3±3.1	0.6162
Total operation time, minutes	316±60	331±30	297±99	0.27
Cardiopulmonary bypass time, minutes	197±48	205±35	187±70	0.4816
Cardiac arrest time, minutes	123±35	120±45	126±34	0.7741

Echocardiography

**Figure 3:**
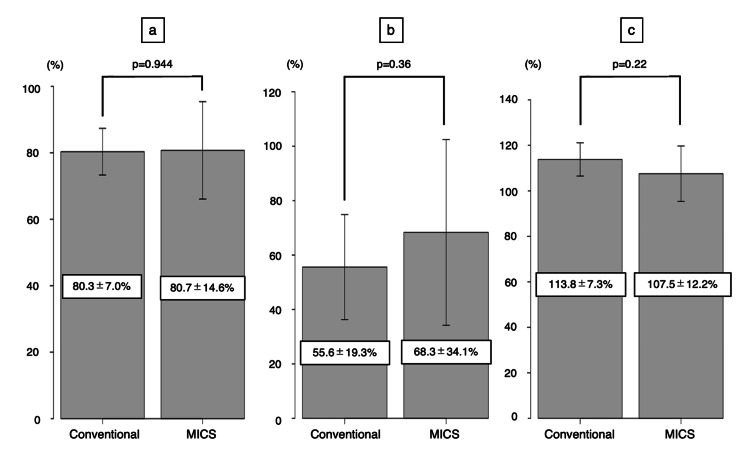
Postoperative echocardiography findings a: Left atrial shrinkage rate. b: Left atrial volume index reduction rate. c: Aorto-mitral angle enlargement rate. p-values less than 0.05 were considered statistically significant. MICS: minimally invasive cardiac surgery

Table 3 displays the preoperative echocardiographic data. No significant divergence was noted in preoperative parameters between Group C and Group M: LA diameter: 54.9±10.8 mm and 56.7±7.3 mm (p=0.68), LAVi: 114.5±38.1 mL/m^2^ and 93.8±79.4 mL/m^2^ (p=0.46), and AMA: 129.6°±15.9° and 136°±3.1° (p=0.17), indicating comparable preoperative profiles. Postoperative assessments revealed analogous efficacy in reducing the LA diameter (C: 80.3±7.0%, M: 80.7±14.6%; p=0.944), LAVi (C: 55.6±19.3%, M: 68.3±34.1%; p=0.36), and AMA enlargement (C: 113.8±7.3%, M: 107.5±12.2%; p=0.22) (Figure 3).

**Table 3:** Preoperative echocardiography findings Data are presented as numbers (%) unless otherwise indicated. p-values less than 0.05 were considered statistically significant. LV: left ventricle, LA: left atrium, MICS: minimally invasive cardiac surgery, Group C: conventional group, Group M: MICS group

	All (n=16)	Conventional group (n=9)	MICS group (n=7)	p-value (Group C versus Group M)
LV end-diastolic dimension, mm	52.6±5.9	52.4±7.2	53.0±6.0	0.222
LV end-systolic dimension, mm	32.9±6.8	31.4±10.7	34.8±4.1	0.3494
LV ejection fraction, %	66.0±11.3	68.4±6.6	62.8±17.8	0.3401
LA dimension, mm	55.7±8.1	54.9±10.8	56.7±7.3	0.6783
LA volume, mL	153.3±83.2	165.9±62.8	137.1±120.4	0.511
LA volume index, mL/m^2^	105.4±53.6	114.5±38.1	93.8±79.4	0.4637
Aorto-mitral angle, °	132.4±9.1	129.6±15.9	136.0±3.1	0.1679

Postoperative outcomes

The postoperative outcomes are described below. No 30-day postoperative or in-hospital mortalities were recorded. Postoperative complications were restricted to singular cases necessitating reoperation due to hemorrhage, albeit originating extraneously in the LA and emerging either from the mediastinum or chest wall. Furthermore, there were no instances of protracted intubation exceeding 48 hours with mechanical ventilation or novel postoperative stroke. The maintenance of sinus rhythm until discharge was confirmed in four (44.4%) and one (14.3%) patients (p=0.308) in Group C and Group M, respectively, with no statistically significant difference noted.

**Figure 4:**
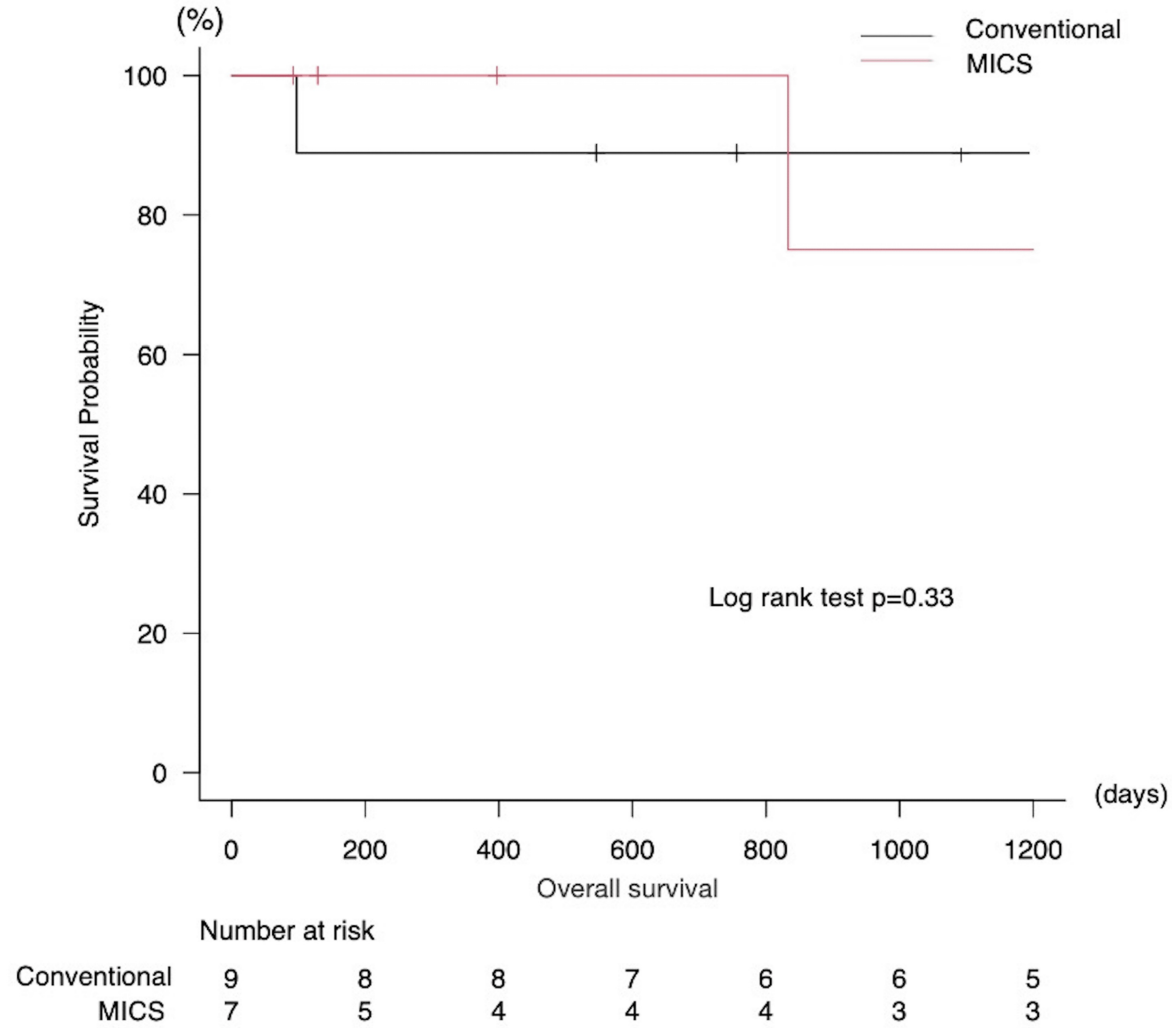
Kaplan-Meier survival curve p-values less than 0.05 were considered statistically significant. MICS: minimally invasive cardiac surgery

The mean duration of follow-up was 3.3±2.4 years. Throughout the observation period, no occurrence of moderate or severe MR, mitral stenosis, or rehospitalization due to heart failure was documented. The three-year survival rate was 88.9% and 75.0% (p=0.33) in Group C and Group M, respectively, with no discernible disparity between the two cohorts (Figure 4). During the observational phase, five mortalities were recorded, none of which were attributable to a cardiac etiology. The causes of death included pneumonia in two cases, stroke in one case, gastrointestinal perforation in one case, and senility in one case.

## Discussion

In this investigation, we present findings concerning the efficacy and safety of LAP with para-annular plication in MICS-MVr via right mini-thoracotomy. Outcomes comparable to those achieved through median sternotomy were observed, notably, a reduction in the LA size. The subsequent discourse will delve into the LAP technique and its efficacy, rooted in the conceivable mechanism of LA enlargement, along with the effectiveness of para-annular plication within the context of MICS-MVr via a right mini-thoracotomy.

LA enlargement is a common observation among individuals with valvular heart disease, particularly in cases of mitral valvular pathology, in which augmentation of the LA dimensions arises from heightened pressure and volume compounded by atrial fibrillation. The anatomical dynamics indicate that while the anterior aspect of the mitral annulus remains relatively stationary, the posterior segment tethered to the posterior LA wall is predisposed to dilation [6,7]. Consequently, enlargement of the LA posterior wall prompts outward deviation of the posterior mitral leaflet, resulting in a reduction in the AMA and potentially leading to functional narrowing of the mitral orifice during diastole [8]. This aberration in AMA dynamics may lead to alterations in LV dynamics and wall shear stress [9]. Notably, studies have posited that postmitral valve surgery AMA constriction correlates with an escalated transmitral pressure gradient and LA strain, thus serving as a potential catalyst for atrial fibrillation [10-12]. Amidst the ongoing debate regarding effective solutions, Kaneyuki et al. [1] underscored the benefits of adjunctive LAP in mitral valve repair.

Despite the ongoing deliberation surrounding the indications, techniques, and efficacy of LAP, the proactive adoption of this approach in mitral valve repair for mitral regurgitation, particularly in cases of LA enlargement (approximate indication: LA diameter > 50 mm), has been advocated by our institution. The para-annular plication technique involves suturing the LA posterior wall between the mitral valve and the right and left inferior pulmonary veins, resulting in postoperative augmentation of the AMA due to the folding of the excess LA tissue. Encouragingly, no instances of postoperative functional mitral stenosis or recurrence of moderate-to-severe MR were documented during the observation period, suggesting favorable outcomes. Regarding postoperative atrial fibrillation, no disparity was noted in the transition to sinus rhythm between the two cohorts. Nevertheless, the potential for inherent bias remains plausible, given that all subjects presented with preoperative atrial fibrillation, albeit with a variable distribution of patients exhibiting either prolonged or indeterminate histories of the condition. Further studies are warranted to discern individualized patient responses based on etiology, necessitating an expansion in case volume.

Recent reports have documented alternative approaches to LAP, characterized by drastic volume reduction techniques involving substantial excision of the LA free wall, including the atrial septum [2,13]. Nonetheless, these techniques are typically reserved for cases with more pronounced LA enlargement (diameter ≥ 70 mm), which differs from our cohort. While such procedures offer promising outcomes, concerns regarding the increased operative complexity, prolonged duration, and associated risks require careful consideration [14,15]. Given the predominantly posterior wall-centric mechanism of LA enlargement, we contend that para-annular plication, tailored to address diameters ranging from 50 to 70 mm, presents a judicious balance between efficacy and procedural safety.

Furthermore, in the burgeoning era of MICS-MVr for MR [4,5], the utility and technique of LAP via right mini-thoracotomy remain unexplored. Our investigation sought to bridge this gap by evaluating the efficacy of LAP with para-annular plication via right mini-thoracotomy. Encouragingly, LAP yielded significant reductions in LA dimensions and volume, indicating favorable postoperative outcomes. Notably, the safety profile of LAP was thoroughly assessed, revealing no discernible disparities in operative or cardiac arrest duration, or ant perioperative complications associated with LAP. Thus, LAP with para-annular plication in MICS-MVr is a promising approach. In the para-annular plication technique, it is imperative to employ continuous suturing with 4-0 monofilament stitches of adequate density to prevent blood leakage, as advocated by Kawazoe et al. [16]. The foremost consideration was to ensure that the suture line maintained an optimal distance from the mitral annulus. This precautionary measure is warranted to avert potential distortion of the mitral valve and mitigate any interference with mitral valve repair. Additionally, meticulous attention must be directed toward needle suturing, as shallow penetration may precipitate inadvertent trauma to the atrial wall tissue, resulting in hematoma formation and hemorrhage. Notably, a hemorrhage stemming from the posterior wall of the LA in the context of a right mini-thoracotomy approach may pose considerable challenges in management, potentially necessitating conversion to a median sternotomy. Conversely, excessive needle thread penetration may cause inadvertent injury to the circumflex branch of the left coronary artery or the esophagus. Fortunately, no such complications were reported at our institution. In summary, while all the aforementioned techniques necessitate scrupulous manipulation, para-annular plication remains a viable approach, even in the context of right mini-thoracotomy.

While acknowledging the merits of this study, it should also be noted that it had some limitations. Owing to the relatively modest sample size and single-center nature of the study, prudence should be exercised when extrapolating the findings. Moreover, inherent biases in case selection, which are typical of retrospective observational studies, cannot be entirely negated. Nonetheless, comparable preoperative patient characteristics between the groups lend credence to the efficacy of the surgical approach. Future studies should prioritize a rigorous evaluation through randomized controlled trials to substantiate these findings.

## Conclusions

In conclusion, this study pioneers the use of the mini-thoracotomy approach for LA plication, presenting a novel technique to the field and demonstrating its unique advantages. The simultaneous application of LAP with para-annular plication in MICS-MVr via right mini-thoracotomy ensures surgical maneuverability and safety equivalent to those observed with median sternotomy. Encouraging outcomes, such as a reduction in LA volume, imply the potential of this technique to enhance postoperative results. When conducting MICS-MVr for cases with LA enlargement of approximately 50-70 mm in diameter, it is advisable to contemplate the concurrent utilization of LAP. Further investigations are imperative to validate the efficacy of LAP as a therapeutic modality for individuals with LA enlargement in future clinical trials.
